# COVID-19 infection in adult patients with hematological malignancies: a European Hematology Association Survey (EPICOVIDEHA)

**DOI:** 10.1186/s13045-021-01177-0

**Published:** 2021-10-14

**Authors:** Livio Pagano, Jon Salmanton-García, Francesco Marchesi, Alessandro Busca, Paolo Corradini, Martin Hoenigl, Nikolai Klimko, Philipp Koehler, Antonio Pagliuca, Francesco Passamonti, Luisa Verga, Benjamin Víšek, Osman Ilhan, Gianpaolo Nadali, Barbora Weinbergerová, Raúl Córdoba-Mascuñano, Monia Marchetti, Graham P. Collins, Francesca Farina, Chiara Cattaneo, Alba Cabirta, Maria Gomes-Silva, Federico Itri, Jaap van Doesum, Marie-Pierre Ledoux, Martin Čerňan, Ozren Jakšić, Rafael F. Duarte, Gabriele Magliano, Ali S. Omrani, Nicola S. Fracchiolla, Austin Kulasekararaj, Toni Valković, Christian Bjørn Poulsen, Marina Machado, Andreas Glenthøj, Igor Stoma, Zdeněk Ráčil, Klára Piukovics, Milan Navrátil, Ziad Emarah, Uluhan Sili, Johan Maertens, Ola Blennow, Rui Bergantim, Carolina García-Vidal, Lucia Prezioso, Anna Guidetti, Maria Ilaria del Principe, Marina Popova, Nick de Jonge, Irati Ormazabal-Vélez, Noemí Fernández, Iker Falces-Romero, Annarosa Cuccaro, Stef Meers, Caterina Buquicchio, Darko Antić, Murtadha Al-Khabori, Ramón García-Sanz, Monika M. Biernat, Maria Chiara Tisi, Ertan Sal, Laman Rahimli, Natasa Čolović, Martin Schönlein, Maria Calbacho, Carlo Tascini, Carolina Miranda-Castillo, Nina Khanna, Gustavo-Adolfo Méndez, Verena Petzer, Jan Novák, Caroline Besson, Rémy Duléry, Sylvain Lamure, Marcio Nucci, Giovanni Zambrotta, Pavel Žák, Guldane Cengiz Seval, Valentina Bonuomo, Jiří Mayer, Alberto López-García, Maria Vittoria Sacchi, Stephen Booth, Fabio Ciceri, Margherita Oberti, Marco Salvini, Macarena Izuzquiza, Raquel Nunes-Rodrigues, Emanuele Ammatuna, Aleš Obr, Raoul Herbrecht, Lucía Núñez-Martín-Buitrago, Valentina Mancini, Hawraa Shwaylia, Mariarita Sciumè, Jenna Essame, Marietta Nygaard, Josip Batinić, Yung Gonzaga, Isabel Regalado-Artamendi, Linda Katharina Karlsson, Maryia Shapetska, Michaela Hanakova, Shaimaa El-Ashwah, Zita Borbényi, Gökçe Melis Çolak, Anna Nordlander, Giulia Dragonetti, Alessio Maria Edoardo Maraglino, Amelia Rinaldi, Cristina De Ramón-Sánchez, Oliver A. Cornely, EPICOVIDEHA working group, Olimpia Finizio, Rita Fazzi, Giuseppe Sapienza, Adrien Chauchet, Jens Van Praet, Juergen Prattes, Michelina Dargenio, Cédric Rossi, Ayten Shirinova, Sandra Malak, Agostino Tafuri, Hans-Beier Ommen, Serge Bologna, Reham Abdelaziz Khedr, Sylvain Choquet, Bertrand Joly, M. Mansour Ceesay, Laure Philippe, Chi Shan Kho, Maximilian Desole, Panagiotis Tsirigotis, Vladimir Otašević, Davimar M. M. Borducchi, Anastasia Antoniadou, Javid Gaziev, Muna A. Almaslamani, Nicole García-Poutón, Giovangiacinto Paterno, Andrea Torres-López, Giuseppe Tarantini, Sibylle Mellinghoff, Stefanie Gräfe, Niklas Börschel, Jakob Passweg, Maria Merelli, Aleksandra Barać, Dominik Wolf, Mohammad Usman Shaikh, Catherine Thiéblemont, Sophie Bernard, Vaneuza Araújo Moreira Funke, Etienne Daguindau, Sofya Khostelidi, Fabio Moore Nucci, Juan-Alberto Martín-González, Marianne Landau, Carole Soussain, Cécile Laureana, Karine Lacombe, Milena Kohn, Gunay Aliyeva, Monica Piedimonte, Guillemette Fouquet, Mayara Rêgo, Baerbel Hoell-Neugebauer, Guillaume Cartron, Fernando Pinto, Ana Munhoz Alburquerque, Juliana Passos, Asu Fergun Yilmaz, Ana-Margarita Redondo-Izal, Fevzi Altuntaş, Christopher Heath, Martin Kolditz, Enrico Schalk, Fabio Guolo, Meinolf Karthaus, Roberta Della Pepa, Donald Vinh, Nicolas Noël, Bénédicte Deau Fischer, Bernard Drenou, Maria Enza Mitra, Joseph Meletiadis, Yavuz M. Bilgin, Pavel Jindra, Ildefonso Espigado, Ľuboš Drgoňa, Alexandra Serris, Roberta Di Blasi, Natasha Ali

**Affiliations:** 1 Hematology, Fondazione Policlinico Universitario Agostino Gemelli - IRCCS – Università Cattolica del Sacro Cuore, Rome, Italy; 2 Università Cattolica del Sacro Cuore, Rome, Italy; 3 Department I of Internal Medicine, Center for Integrated Oncology Aachen Bonn Cologne Duesseldorf, Excellence Center for Medical Mycology (ECMM), University of Cologne, Faculty of Medicine and University Hospital Cologne, Cologne, Germany; 4 Cologne Excellence Cluster On Cellular Stress Responses in Aging-Associated Diseases (CECAD), University of Cologne, Faculty of Medicine and University Hospital Cologne, Cologne, Germany; 5 Hematology and Stem Cell Transplant Unit, IRCCS Regina Elena National Cancer Institute, Rome, Italy; 6 Stem Cell Transplant Center, AOU Citta’ Della Salute E Della Scienza, Turin, Italy; 7 University of Milan and Fondazione IRCCS Istituto Nazionale Dei Tumori, Milan, Italy; 8 Division of Infectious Diseases and Global Public Health, Department of Medicine, University of California San Diego, San Diego, CA USA; 9 Clinical and Translational Fungal-Working Group, University of California San Diego, La Jolla, CA USA; 10 Section of Infectious Diseases and Tropical Medicine, Department of Internal Medicine, Medical University of Graz, Graz, Austria; 11 North-Western State Medical University Named After Iliá Ilich Méchnikov, Saint-Petersburg, Russia; 12 Department of Hematological Medicine, King’s College Hospital NHS Foundation Trust, London, UK; 13 Department of Medicine and Surgery, University of Insubria, ASST Sette Laghi, Ospedale Di Circolo of Varese, Varese, Italy; 14 Azienda Ospedaliera San Gerardo - Monza, Monza, Italy; 15 Università Milano-Bicocca, Milan, Italy; 16 University Hospital Hradec Králové, Hradec Králové, Czech Republic; 17 Ankara University, Ankara, Turkey; 18 Policlinico Borgo Roma Verona, Verona, Italy; 19 Department of Internal Medicine, Hematology and Oncology, Masaryk University and University Hospital Brno, Brno, Czech Republic; 20 Fundacion Jimenez Díaz University Hospital, Health Research Institute IIS-FJD, Madrid, Spain; 21 Hematology and BMT Unit, Azienda Ospedaliera Nazionale SS. Antonio E Biagio E Cesare Arrigo, Alessandria, Italy; 22 NIHR Oxford Biomedical Research Centre, Churchill Hospital, Oxford, UK; 23 Hematology and Bone Marrow Transplantation, IRCCS San Raffaele Scientific Institute, Milan, Italy; 24 ASST-Spedali Civili, Brescia, Italy; 25 Department of Hematology, Vall d’Hebron Hospital Universitari, Experimental Hematology, Vall d’Hebron Institute of Oncology (VHIO), Vall d’Hebron Barcelona Hospital Campus, Barcelona, Spain; 26 Departament de Medicina, Universitat Autònoma de Barcelona, Bellaterra, Spain; 27 Portuguese Institute of Oncology, Lisbon, Portugal; 28 San Luigi Gonzaga Hospital - Orbassano, Orbassano, Italy; 29 University Medical Center Groningen, Groningen, Netherlands; 30 ICANS, Strasbourg, France; 31 Department of Hemato-Oncology, Faculty of Medicine and Dentistry, Palacky University and University Hospital Olomouc, Olomouc, Czech Republic; 32 Department of Hematology, University Hospital Dubrava, Zagreb, Croatia; 33 Hospital Universitario Puerta de Hierro, Majadahonda, Spain; 34 ASST Grande Ospedale Metropolitano Niguarda, Milan, Italy; 35 Communicable Disease Center, Hamad Medical Corporation, Doha, Qatar; 36 Fondazione IRCCS Ca’ Granda Ospedale Maggiore Policlinico, Milan, Italy; 37 King’s College Hospital, London, UK; 38 King’s College London, London, UK; 39 University Hospital Centre Rijeka, Rijeka, Croatia; 40 Croatian Cooperative Group for Hematological Diseases (CROHEM), Zagreb, Croatia; 41 Faculty of Medicine and Faculty of Health Studies, University of Rijeka, Rijeka, Croatia; 42 Zealand University Hospital, Roskilde, Roskilde, Denmark; 43 Clinical Microbiology and Infectious Diseases Department, Hospital General Universitario Gregorio Marañón, Madrid, Spain; 44 Rigshospitalet, Copenhagen, Denmark; 45 Gomel State Medical University, Gomel, Belarus; 46 Institute of Hematology and Blood Transfusion, Prague, Czech Republic; 47 Department of Internal Medicine, Albert Szent-Györgyi Health Center, Faculty of Medicine, University of Szeged, Szeged, Hungary; 48 University Hospital Ostrava, Ostrava, Czech Republic; 49 Oncology Center, Mansoura University, Mansoura, Egypt; 50 Marmara University, Istanbul, Turkey; 51 KU Leuven, Leuven, Belgium; 52 Department of Infectious Diseases, Karolinska University Hospital, Stockholm, Sweden; 53 3S-Instituto de Investigação E Inovação Em Saúde, Universidade Do Porto, Porto, Portugal; 54 Cancer Drug Resistance Group, IPATIMUP-Institute of Molecular Pathology and Immunology, University of Porto, Porto, Portugal; 55 Clinical Hematology, Centro Hospitalar E Universitário São João, Porto, Portugal; 56 Clinical Hematology, Faculty of Medicine, University of Porto, Porto, Portugal; 57 Hospital Clinic, Barcelona, Spain; 58 U.O. Ematologia E Centro Trapianti Midollo Osseo, Ospedale Maggiore, Parma, Italy; 59 Fondazione IRCCS Istituto Nazionale Dei Tumori, Milan, Italy; 60 Department of Biomedicine and Prevention, University of Rome Tor Vergata, Rome, Italy; 61 Hematology and Transplantation, Raisa Gorbacheva Research Institute of Pediatric Oncology, Pavlov University, St. Petersburg, Russia; 62 Amsterdam UMC, Location VUmc, Amsterdam, Netherlands; 63 Complejo Hospitalario de Navarra, Iruña-Pamplona, Spain; 64 Hospital Universitario Marqués de Valdecilla, Santander, Spain; 65 La Paz University Hospital, Madrid, Spain; 66 Hematology Unit, Center for Translational Medicine, Azienda USL Toscana NordOvest, Livorno, Italy; 67 AZ Klina, Brasschaat, Belgium; 68 Ematologia Con Trapianto, Ospedale Dimiccoli Barletta, Barletta, Italy; 69 Clinic of Hematology, University Clinical Center of Serbia, Belgrade, Serbia; 70 Faculty of Medicine, University of Belgrade, Belgrade, Serbia; 71 Sultan Qaboos University Hospital, Muscat, Oman; 72 Hematology Department, Hospital Universitario de Salamanca, Salamanca, Spain; 73 IBSAL, Centro de Investigación del Cáncer-IBMCC (USAL-CSIC), Salamanca, Spain; 74 Department of Haematology, Blood Neoplasms, and Bone Marrow Transplantation, Wroclaw Medical University, Wrocław, Poland; 75 Cell Therapy and Hematology, San Bortolo Hospital, Vicenza, Italy; 76 Department of Oncology, Hematology and Bone Marrow Transplantation With Section of Pneumology, University Medical Center Hamburg-Eppendorf, Hamburg, Germany; 77 Hospital Universitario 12 de Octubre, Madrid, Spain; 78 Azienda Sanitaria Universitaria del Friuli Centrale, Udine, Italy; 79 Hospital Rey Juan Carlos, Móstoles, Spain; 80 Division of Infectious Diseases and Hospital Epidemiology, Department of Clinical Research, University and University Hospital of Basel, Basel, Switzerland; 81 Hospital Escuela de Agudos Dr. Ramón Madariaga, Posadas, Argentina; 82 Department of Hematology and Oncology, Medical University of Innsbruck, Innsbruck, Austria; 83 University Hospital of Královské Vinohrady, Prague, Czech Republic; 84 Centre Hospitalier de Versailles, Versailles, France; 85 Service d’Hématologie Clinique Et de Thérapie Cellulaire, Hôpital Saint Antoine, Assistance Publique-Hôpitaux de Paris, Sorbonne Université, Inserm UMRs 938, Paris, France; 86 Departement d’Hematologie Clinique, CHU de Montpellier, UMR-CNRS 5535, Universite de Montpellier, Montpellier, France; 87 Federal University of Rio de Janeiro, Rio de Janeiro, Brazil; 88 Fundacion Jimenez Diaz University Hospital, Health Research Institute IIS-FJD, Madrid, Spain; 89 National Center for Cancer Care and Research, Hamad Medical Corporation, Doha, Qatar; 90 University Hospital Centre Zagreb, Zagreb, Croatia; 91 Faculty of Medicine, University of Zagreb, Zagreb, Croatia; 92 Hematology Service, Instituto Nacional Do Cancer, Rio de Janeiro, Brazil; 93 Haematology and Haemotherapy Department, Hospital General Universitario Gregorio Marañón, Madrid, Spain; 94 Scientific and Practical Center for Surgery, Transplantology and Hematology, Minsk, Belarus; 95 Department of Cell Therapy and Allogenic Stem Cell Transplantation (CAST), Karolinska University Hospital, Stockholm, Sweden; 96 Centro de Investigación del Cáncer-IBMCC (USAL-CSIC), Hospital Universitario de Salamanca, IBSAL, Salamanca, Spain; 97 Chair Translational Research, Cologne Excellence Cluster On Cellular Stress Responses in Aging-Associated Diseases (CECAD), University of Cologne, Faculty of Medicine and University Hospital Cologne, Cologne, Germany; 98 Clinical Trials Centre Cologne (ZKS Köln), University of Cologne, Faculty of Medicine and University Hospital Cologne, Cologne, Germany; 99 Center for Molecular Medicine Cologne (CMMC), University of Cologne, Faculty of Medicine and University Hospital Cologne, Cologne, Germany; 100 German Centre for Infection Research (DZIF), Partner Site Bonn-Cologne, Cologne, Germany

**Keywords:** COVID-19, Pandemic, Hematological malignancies, Epidemiology, EHA

## Abstract

**Background:**

Patients with hematological malignancies (HM) are at high risk of mortality from SARS-CoV-2 disease 2019 (COVID-19). A better understanding of risk factors for adverse outcomes may improve clinical management in these patients. We therefore studied baseline characteristics of HM patients developing COVID-19 and analyzed predictors of mortality.

**Methods:**

The survey was supported by the Scientific Working Group Infection in Hematology of the European Hematology Association (EHA). Eligible for the analysis were adult patients with HM and laboratory-confirmed COVID-19 observed between March and December 2020.

**Results:**

The study sample includes 3801 cases, represented by lymphoproliferative (mainly non-Hodgkin lymphoma *n* = 1084, myeloma *n* = 684 and chronic lymphoid leukemia *n* = 474) and myeloproliferative malignancies (mainly acute myeloid leukemia *n* = 497 and myelodysplastic syndromes *n* = 279). Severe/critical COVID-19 was observed in 63.8% of patients (*n* = 2425). Overall, 2778 (73.1%) of the patients were hospitalized, 689 (18.1%) of whom were admitted to intensive care units (ICUs). Overall, 1185 patients (31.2%) died. The primary cause of death was COVID-19 in 688 patients (58.1%), HM in 173 patients (14.6%), and a combination of both COVID-19 and progressing HM in 155 patients (13.1%). Highest mortality was observed in acute myeloid leukemia (199/497, 40%) and myelodysplastic syndromes (118/279, 42.3%). The mortality rate significantly decreased between the first COVID-19 wave (March–May 2020) and the second wave (October–December 2020) (581/1427, 40.7% vs. 439/1773, 24.8%, *p* value < 0.0001). In the multivariable analysis, age, active malignancy, chronic cardiac disease, liver disease, renal impairment, smoking history, and ICU stay correlated with mortality. Acute myeloid leukemia was a higher mortality risk than lymphoproliferative diseases.

**Conclusions:**

This survey confirms that COVID-19 patients with HM are at high risk of lethal complications. However, improved COVID-19 prevention has reduced mortality despite an increase in the number of reported cases.

**Supplementary Information:**

The online version contains supplementary material available at 10.1186/s13045-021-01177-0.

## Background

Coronavirus disease 19 (COVID-19), caused by severe acute respiratory syndrome coronavirus 2 (SARS-CoV-2) was declared a pandemic by the World Health Organization (WHO) in March 2020 [1]. During that year, COVID-19 spread worldwide, causing over 1.5 million deaths. Patients with hematological malignancies (HM) are considered at high risk of developing severe and life-threatening infections, because of immune deficiency and immunosuppressive treatments. Severe infections in HM patients can determine a worsening of the clinical outcome, potentially affecting life expectancy. SARS-CoV-2 affects HM patients disproportionally, leading often to severe COVID-19 with a high mortality rate [2]. So far, various reports have been published on COVID-19 HM patients, but in most cases on small patient cohorts [3–8], specific HM [9–12], or larger reports from single countries [13–16]. In June 2021, an ongoing world-wide registry of the American Society of Hematology (ASH) reported a total of 1013 cases of COVID-19 infections in HM [17]. Altogether, these data show a significant mortality rate, ranging between 13.8 and 39%, and highlighting the major relevance of COVID-19 management in this frail patient population [3–17]. Advanced disease, one or more co-morbidities, older age, type of malignancy, in particular acute myeloid leukemia (AML), and several laboratory parameters, for example high C-reactive protein, lymphopenia, and neutropenia, were found to be risk factors for COVID-19 in HM patients [14–16]. A possible role of some antineoplastic drugs has been reported to be protective in patients with myeloproliferative disorders [18, 19]. Despite the current spread of vaccination programs among HM patients in several countries, the future trajectory of this pandemic seems still to be uncertain. Collecting further data and gaining a better knowledge about COVID-19 in HM is therefore relevant for hematologists around the world.

The EPICOVIDEHA, Epidemiology of COVID-19 Infection in Patients with Hematological Malignancies: A European Hematology Association Survey, multinational project aimed to collect COVID-19 cases occurring in HM patients in 2020, and was performed on behalf of the Scientific Working Group Infection in Hematology of the European Hematology Association (EHA). The objective was to assess epidemiology and outcomes of COVID-19 in HM patients.

## Methods

### Study design and patients

EPICOVIDEHA is an international open web-based registry for patients with HM infected with SARS-CoV-2 [20]. The survey has been approved by the Institutional Review Board and Ethics Committee of the Fondazione Policlinico Universitario Agostino Gemelli—IRCCS, Università Cattolica del Sacro Cuore of Rome, Italy (Study ID: 3226). The corresponding local ethics committee of each participating institution has approved the EPICOVIDEHA study when applicable. EPICOVIDEHA has been registered at www.clinicaltrials.gov with the identifier NCT04733729. Different medical hematology societies have joined this project (Additional file 1: Table 1). Participating institutions documented episodes of COVID-19 in their patients with baseline HM between March 2020 and December 2020. Data were collected via the EPICOVIDEHA electronic case report form (eCRF), available at www.clinicalsurveys.net. This online survey is provided by EFS Fall 2018 (Questback, Cologne, Germany).

### Procedures

Experts at the University Hospital Cologne, Cologne, Germany, with previous experience in the research and study of HM and infectious diseases, reviewed each case included in the registry, for completeness and consistency. Each patient was reviewed for validity following the inclusion criteria: (a) HM (excluding non-malignant hematological disorders or solid tumors), (b) malignancy with activity during the 5 years before COVID-19 (either diagnosis or treatment), (c) patient over 18 years of age, (d) hematological diagnosis before COVID-19, and (e) laboratory diagnosis for COVID-19 (not clinical diagnosis). Data on patients’ demographic characteristics and baseline conditions before COVID-19 were collected. Additional variables, such as type of COVID-19 test, the reason for COVID-19 test, admission to ICU after COVID-19, day of death, and cause of death were collected.

The diagnosis of COVID-19 was made according to the international recommendations of the WHO [21]. At the time of the survey design, no well-defined criteria were yet available to establish a degree of infection severity. Therefore, the following definitions have been included: asymptomatic (no clinical signs or symptoms); mild (non-pneumonia and mild pneumonia); severe (dyspnea, respiratory frequency ≥ 30 breaths per min, SpO2 ≤ 93%, PaO_2_/FiO_2_ < 300, or lung infiltrates > 50%), and critical (patients admitted in intensive care for respiratory failure, septic shock, or multiple organ dysfunction or failure). However, our grading definition was very similar to the one suggested by the China Centers for Disease Control and Prevention definitions [22]. Overall case-fatality rate (overall mortality) was define as the proportion of deaths for any cause compared to the total number of patients registered during the observation time. Attributable or contributable deaths were defined on the basis of subjective judgment of the local physician.

### Study objectives

The primary objective of this study was to assess the epidemiology and the outcome of HM affected by COVID-19. Secondary objectives were: (1) to estimate the prevalence of disease severity (i.e., asymptomatic, mild, severe disease); (2) to evaluate the prevalence of ICU admission; (3) to estimate the frequency of pre-existing co-morbidities; (4) to evaluate the overall case-fatality rate; (5) to assess geographical patterns of the disease; (6) to stratify patients according to treatment of the underlying HM (off/on) and according to type of therapy (i.e., chemotherapy, immunotherapy, targeted therapy, hematopoietic stem cell transplant [HSCT]).

### Statistical analysis

The primary analysis describes the demographic and clinical characteristics of patients with COVID-19 after a previous HM diagnosis. Categorical variables are presented with frequencies and percentages, and continuous variables with median, interquartile range (IQR) and absolute range. The secondary analysis studies independent predictors of overall mortality in hematological patients with COVID-19, by employing a Cox proportional hazard model. Univariable Cox regression model was performed with variables suspected to play a role in the mortality of HM patients with COVID-19 (i.e. sex [reference female], age, malignancy status [reference controlled disease], hematological malignancy [reference Hodgkin lymphoma], COVID-19 infection [reference asymptomatic], ICU stay, chronic cardiopathy, liver disease, chronic pulmonary disease, diabetes mellitus, obesity, renal impairment, smoking history, neutrophils [reference ≤ 500 units/mm^3^], lymphocytes [reference ≤ 200 units/mm^3^], and last chemotherapy [reference > 3 months before COVID-19]). Variables with a p-value ≤ 0.1 were considered for multivariable analysis. A multivariable Cox regression model was calculated with the Wald backward method, and only those variables that were statistically significant displayed. Mortality was analyzed using Kaplan–Meier survival plots. Log-rank test was used to compare the survival probability of the patients included in the different models, based on COVID-19 severity, baseline malignancy, pandemic wave, and HSCT/non-HSCT. A p-value ≤ 0.05 was considered statistically significant. No a priori sample size calculation was done for this exploratory study. SPSSv25.0 was employed for statistical analyses (SPSS, IBM Corp., Chicago, IL, United States).

### Role of funding source

The funder of the study had no role in study design, data analysis, and interpretation, or writing of the report. All authors had full access to the data and had final responsibility for the decision to submit for publication.

## Results

**Fig. 1:**
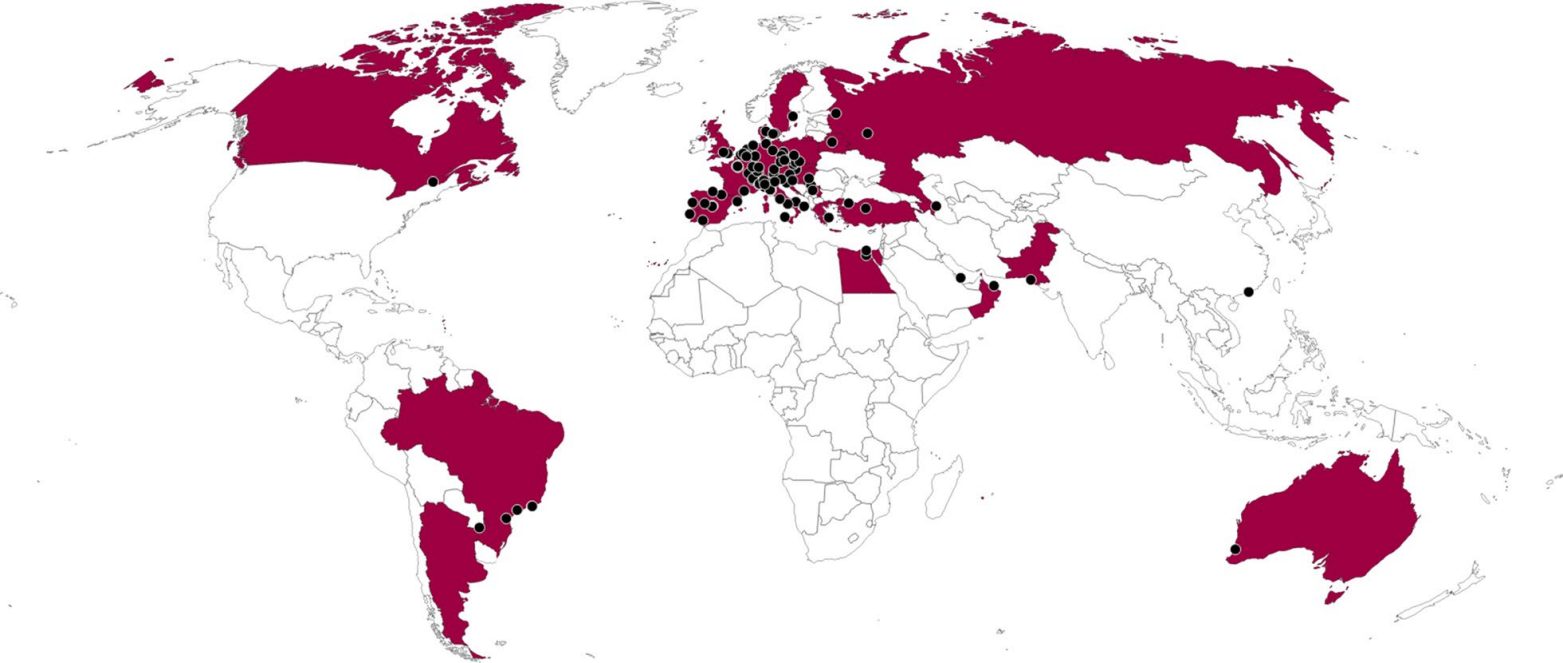
Geographical distribution of patient reported to EPICOVIDEHA

A total of 132 centers in 32 countries participated in this survey (Fig. 1, Additional file 2: Tables 2), and registered 4117 cases. Of these, 316 (7.7%) were excluded for the following reasons: age < 18 years old, clinical diagnosis of COVID-19, double-entry, non-malignant hematological diseases, incomplete information, more than 5 years off-therapy from the last chemotherapy, or solid cancer.

The demographic and clinical characteristics of 3801 valid cases are reported in Table 1. There was a higher prevalence of males (*n* = 2222, 58.5%) and Caucasian ethnic background (*n* = 3289, 86.5%). The median age was 65 years (IQR: 54–74; range 18–95).

**Table 1:** Demographic and clinical characteristics of enrolled patients at COVID-19 diagnosis

	*n*	%
*Sex*		
Female	1579	41.5
Male	2222	58.5
Age, median (IQR) [range]	65 (54–74), [18–95]	
*Comorbidities*		
Chronic cardiopathy	1146	30.1
Chronic pulmonary disease	614	16.2
Diabetes mellitus	620	16.3
Liver disease	167	4.4
Obesity	345	9.1
Renal impairment	325	8.6
Smoking history	477	12.5
No risk factor identified	1463	38.5
*Baseline hematological malignancies*		
Acute lymphoid leukemia	169	4.4
Chronic lymphoid leukemia	474	12.5
Acute myeloid leukemia	497	13.1
Chronic myeloid leukemia	161	4.2
Myelodysplastic syndrome	279	7.3
Low-intermediate risk	138	3.6
High risk	48	1.3
Not stated	93	2.4
Hairy cell leukemia	23	0.6
Hodgkin lymphoma	135	3.6
Non-Hodgkin lymphoma	1084	28.5
Indolent	497	13.1
Aggressive	516	13.6
Not stated	71	1.9
Essential thrombocythemia	69	1.8
Myelofibrosis	122	3.2
Polycythemia vera	70	1.8
Systemic mastocytosis	6	0.2
Multiple myeloma	684	18.0
Amyloidosis	8	0.2
Aplastic anemia	20	0.5
*Status*^*a*^		
Controlled disease	1760	46.3
Complete remission	1170	30.8
Partial remission	590	15.5
Active disease	1963	51.6
Onset	888	23.4
Refractory/Resistant	473	12.4
Stable disease	524	13.8
Unknown	78	2.1
*Unknown*	78	2.1

Data can be super additive

^a^Onset patients had a contemporaneous diagnosis of the malignancy and the COVID-19, regardless of malignancy treatment initiation. Stable disease patients include patients at watch and wait

Patients with non-Hodgkin lymphoma (NHL) represented the largest subgroup (*n* = 1084, 28.5%), followed by patients with multiple myeloma (MM) (*n* = 684, 18%) and those with AML (*n* = 497, 13.1%). Overall, 67.3% of the patients who developed COVID-19 had a baseline lymphoproliferative disease (*n* = 2557) (Table 1). More than 51% of the patients had active disease (*n* = 1963), and 2502 patients (65.8%) had received chemotherapy in the 3 months before the onset of COVID-19 (Table 2). The most frequent treatments were chemotherapy with immunotherapy or immunotherapy alone administered to 983 patients (25.9%), compatible with the proportion of patients with NHL. In 271 patients (7.1%), the infection occurred concomitantly with the diagnosis of HM, and in 138 of those (50.9%) before treatment initiation for the baseline malignancy (Table 1). Five hundred fifty-seven patients (14.7%) had a transplant procedure performed in their clinical history (292 autologous HSCT [auto-HSCT] and 265 allogeneic HSCT [allo-HSCT]). In 247 patients, 173 allo-HSCT and 74 auto-HSCT recipients, the transplant procedure was the last therapy before COVID-19 infection. In total 24 patients in the registry were treated with chimeric antigen receptor T (CAR-T) cells reinfusion, of which 3 patients had been treated with additional therapies after the CAR-T cell therapy (Table 2).

**Table 2:** Summary of received treatments for Hematological Malignancies at the onset of COVID-19

	*n*	%
*Last/ongoing treatment strategy before COVID-19*		
Immunochemotherapy	857	22.5
Targeted therapy^a^	607	16.0
Conventional chemotherapy	597	15.7
No treatment	538	14.1
Palliative/supportive measures	226	6.0
Immunomodulators	218	5.7
Allogeneic HSCT	173	4.6
Anagrelide/Hydroxyurea	145	3.8
Hypomethylating agents	141	3.7
Immunotherapy only	125	3.3
Autologous HSCT	74	1.9
Unknown	41	1.1
Other	28	0.7
CAR-T	21	0.6
Radiotherapy	10	0.3
*Summary of received treatment*^b^		
Chemotherapy	3178	83.6
In the last month	1979	52.1
In the last 3 months	523	13.8
Treatment ended > 3 months	631	16.6
Not stated	45	1.2
Radiotherapy	186	4.9
Allogeneic HSCT	265	7.0
Autologous HSCT	292	7.7
CAR-T	24	0.6
Other strategies	150	3.9
No treatment	538	14.2

*HSCT* Hematopoietic stem cell transplantation, *CAR-T* chimeric antigen receptor T-cell therapies

^a^Bortezomib, ibrutinib, idelalisib, ruxolitinib, TKI (tyrosine kinase inhibitors), and venetoclax

^b^Data can be super-additive

Overall, 2304 (60.6%) patients had at least one comorbidity, with cardiovascular diseases being most frequent (*n* = 1146, 30.1%). In 447 patients (12.5%) smoking history was reported (Table 1).

At the onset of COVID-19 infection, 280 patients (7.4%) had neutrophils below 0.5 × 10^9^/mm^3^, and 344 patients (9.1%) lymphocytes below 0.2 × 10^9^/mm^3^ (Table 3).

**Table 3:** Clinical features of COVID-19 in our patient cohort

	*n*	%
*COVID-19 infection*		
Asymptomatic	675	17.8
Mild	658	17.3
Severe	1736	45.7
Critical	689	18.1
Unknown	43	1.1
*COVID-19 test sample*^a^		
BAL	60	1.6
SARS-CoV-2 nasopharyngeal swab	3700	97.3
SARS-CoV-2 serology	86	2.3
*Reason for COVID-19 test*^a^		
Pulmonary symptoms	1454	38.3
Pulmonary + extrapulmonary symptoms	831	21.9
Extrapulmonary symptoms	742	19.5
Screening	727	19.1
Unknown	47	1.2
*Neutrophils level at COVID-19 diagnosis*^b^		
≤ 0.5 × 10^9^/mm^3^	280	7.4
0.501–0.999 × 10^9^/mm^3^	217	5.7
≥ 1 × 10^9^/mm^3^	2738	72.0
*Lymphocytes level at COVID-19 diagnosis*^b^		
≤ 0.2 × 10^9^/mm^3^	344	9.1
0.201–0.499 × 10^9^/mm^3^	538	14.2
≥ 0.5 × 10^9^/mm^3^	2367	62.3
*Stay during COVID-19*		
Admitted to hospital	2778	73.1
Length of hospital stay, median (IQR) [range]	15 (8–27), [1–235]	–
ICU	689	18.1
Length of ICU stay, median (IQR) [range]	11 (5–20), [1–111]	–
Invasive MV	449	11.8
Non-invasive MV	221	5.8
*Clinical outcome of COVID-19*		
Death	1185	31.2
Observation time, median (IQR) [range]	89 (21–172), [0–436]	–
*Reason for death*^a^		
Not related to COVID-19	125	3.3
Contributable by COVID-19	155	4.1
Attributable to COVID-19	843	22.2
Attributable to HM	328	8.6
Death due to other reasons	123	3.2
Death due to unknown reasons	78	2.1

*BAL* Bronchoalveolar lavage, *COVID-19* coronavirus disease 19, *HM* hematological malignancy, *ICU* intensive care unit, *MV* mechanical ventilation, *SARS-CoV-2* severe acute respiratory syndrome coronavirus 2

^a^Data can be super additive

^b^Data not available in all patients

SARS-CoV-2 infection was diagnosed by nasopharyngeal swab in almost all patients (*n* = 3700, 97.3%). COVID-19 tests were performed in 3027 patients (79.6%) because of pulmonary and/or extrapulmonary symptoms, and in 727 patients (19.1%) as part of asymptomatic screening. Reason for testing was unknown in 47 (1.2%). Presence of respiratory symptoms, mainly cough and dyspnea, was the most frequent clinical presentation, reported in 2285 (60.1%), and in 831 of them (21.9%) it was combined with extra-pulmonary symptoms. In 742 patients (19.5%) extra-pulmonary symptoms, in particular anosmia, diarrhea, skin rash, were predominant in terms of clinical presentation (Table 3).

COVID-19 infection was determined to be critical in 689 patients (18.1%), severe in 1736 (45.7%), mild in 658 (17.3%), and asymptomatic in 675 (17.8%) (Table 3).

Overall, 2778 patients (73.1%) were hospitalized. The median duration of overall hospitalization was 15 days (IQR: 8–27, range 1–235), regardless of patient outcome. Among the hospitalized patients, 689 (18.1%) required hospitalization in an ICU, 449 of these (65.2%) with invasive mechanical ventilation (MV) (Table 3).

Altogether, during the observation phase, 1185 patients (31.2%) died. The primary cause of death was COVID-19 in 688 patients (58.1%), HM in 173 patients (14.6%), and a combination of both COVID-19 and progressing HM in 155 patients (13.1%). In the remaining cases the cause was unknown or due to other reasons.

**Fig. 2:**
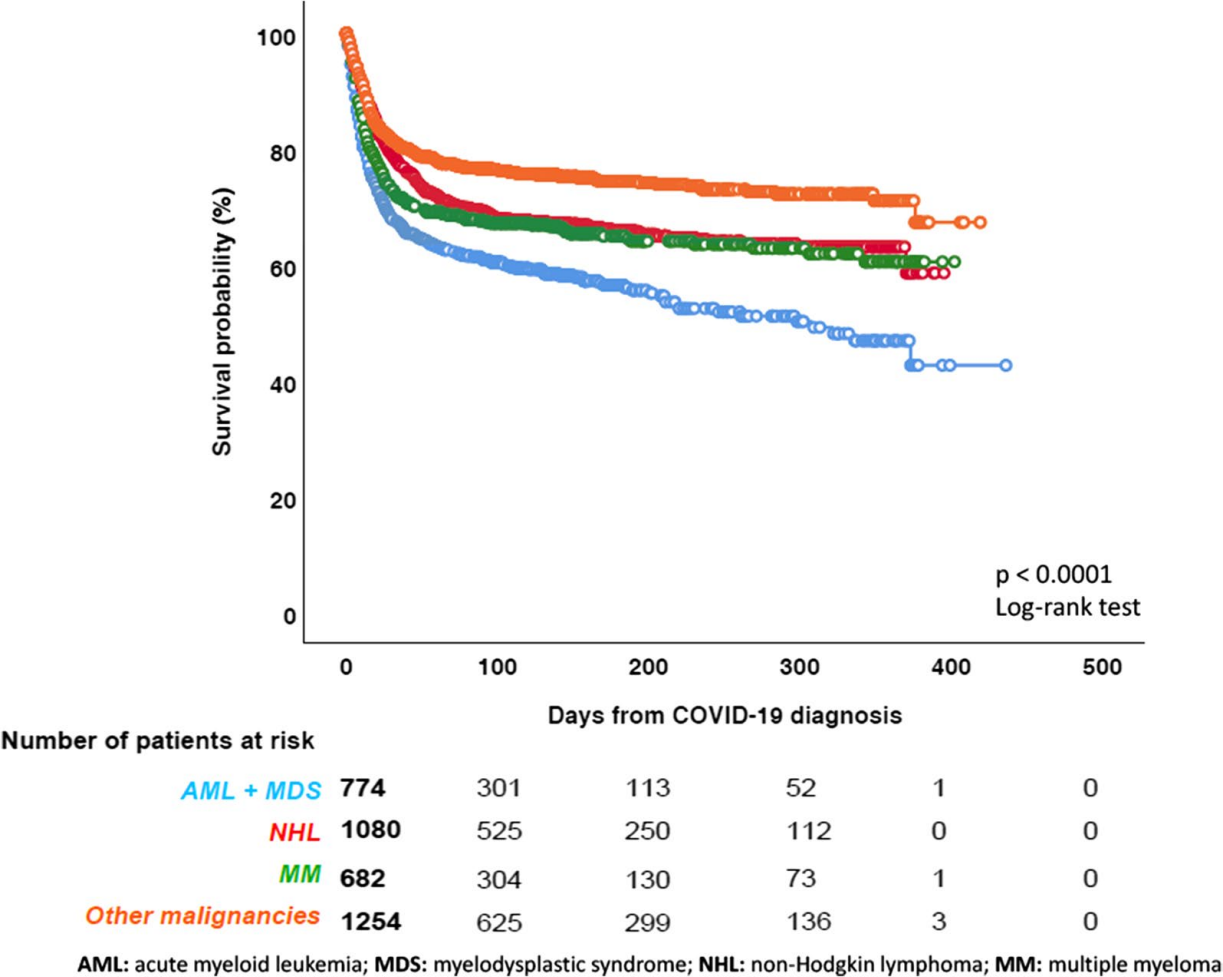
Overall survival by the underlying disease

Patients over the age of 70 years had the highest mortality (661/1475, 44.8%). Considering the different HM, the higher number of fatalities was observed in AML (199/497, 40%) and in myelodysplastic syndromes (MDS) (118/279, 42.3%) (Table 4). Mortality in AML/MDS was significantly higher when compared to mortality in other HM (*p* < 0.0001) (Fig. 2).

**Table 4:** Overall mortality rate by disease and treatment received

	Overall mortality	
	Survived *n* (%)	Died *n* (%)
*Baseline hematological malignancies*		
Acute lymphoid leukemia	125 (74)	44 (26)
Chronic lymphoid leukemia	340 (71.7)	134 (28.3)
Acute myeloid leukemia	298 (60)	199 (40)
Chronic myeloid leukemia	144 (89.5)	17 (10.5)
Myelodysplastic syndrome	161 (57.7)	118 (42.3)
Low-intermediate risk	77 (55.8)	61 (44.2)
High risk	26 (54.2)	22 (45.8)
Not stated	58 (62.4)	35 (37.6)
Hairy cell leukemia	15 (65.2)	8 (34.8)
Hodgkin lymphoma	120 (88.9)	15 (11.1)
Non-Hodgkin lymphoma	739 (68.2)	345 (31.8)
Indolent	354 (71.3)	143 (28.7)
Aggressive	337 (65.3)	179 (34.7)
Not stated	48 (67.6)	23 (32.4)
Essential thrombocythemia	57 (82.6)	12 (17.4)
Myelofibrosis	77 (63.1)	45 (36.9)
Polycythemia vera	56 (80)	14 (20)
Systemic mastocytosis	5 (83.4)	1 (16.6)
Multiple Myeloma	458 (67)	226 (33)
Amyloidosis	7 (87.5)	1 (12.5)
Aplastic anemia	14 (70)	6 (30)
*Last/ongoing treatment strategy before COVID-19*		
Anagrelide/Hydroxyurea	106 (73.1)	39 (26.9)
Conventional chemotherapy	423 (70.9)	174 (29.1)
Hypomethylating agents	58 (41.2)	83 (58.8)
Immunotherapy only	89 (71.2)	36 (28.8)
Immunochemotherapy	595 (69.4)	262 (30.6)
Immunomodulators	139 (63.8)	79 (36.2)
Targeted therapy^a^	453 (74.6)	154 (25.4)
Allogeneic HSCT	130 (75.2)	43 (24.8)
Autologous HSCT	54 (73)	20 (27)
CAR-T	11 (52.4)	10 (47.6)
Radiotherapy	9 (90)	1 (10)
Palliative/supportive measures	122 (56)	104 (46)
Other	17 (60.7)	11 (39.3)
Unknown	28 (68.3)	13 (31.7)
No treatment	382 (71)	156 (29)

*HSCT* Hematopoietic stem cell transplantation, *CAR-T* chimeric antigen receptor T-cell therapies

^a^Bortezomib, ibrutinib, idelalisib, ruxolitinib, TKI (tyrosine kinase inhibitors) and venetoclax

**Fig. 3:**
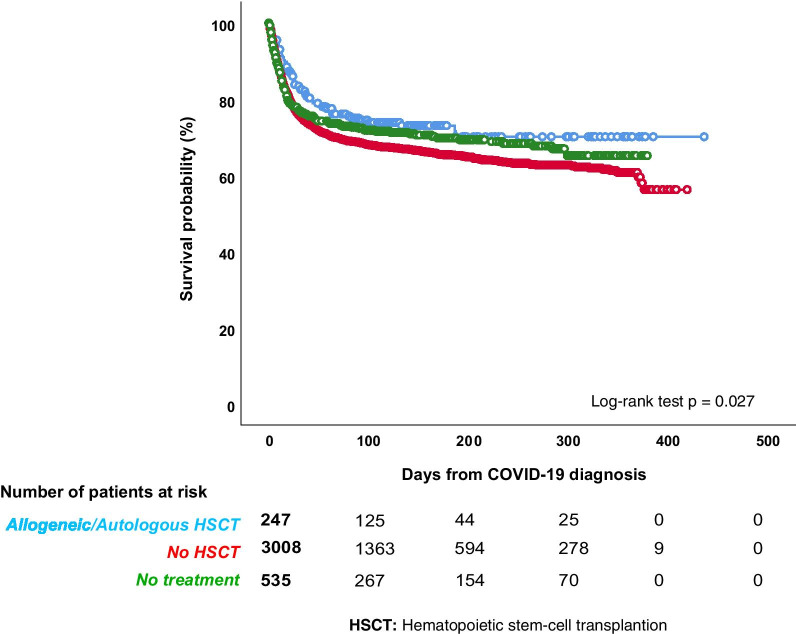
Overall survival by transplant *vs* no transplant

Regarding last underlying treatments for HM before COVID-19, the highest mortality rate was observed among patients receiving demethylating agents (83/141, 58.9% [95% confidence interval {CI} 50.6–66.7]) and in palliative treatment settings (104/226, 46% [95%CI 39.4–52.5]). Despite the small number of patients undergoing CAR-T reinfusion, mortality rate in these patients was high (47.6% [95% CI 28.3–67.6]; 10/21 patients). Patients undergoing auto-HSCT or allo-HSCT had mortality rates of 27% ([95% CI 18.2–38.1] 20/74 cases) and 24.8% ([95% CI 19.0–31.8] 43/173 cases), respectively (Table 4). The mortality rate of patients who received a transplant as most recent therapy was significantly lower when compared to non-transplant patients (*p* < 0.027) (Fig. 3).

**Fig. 4:**
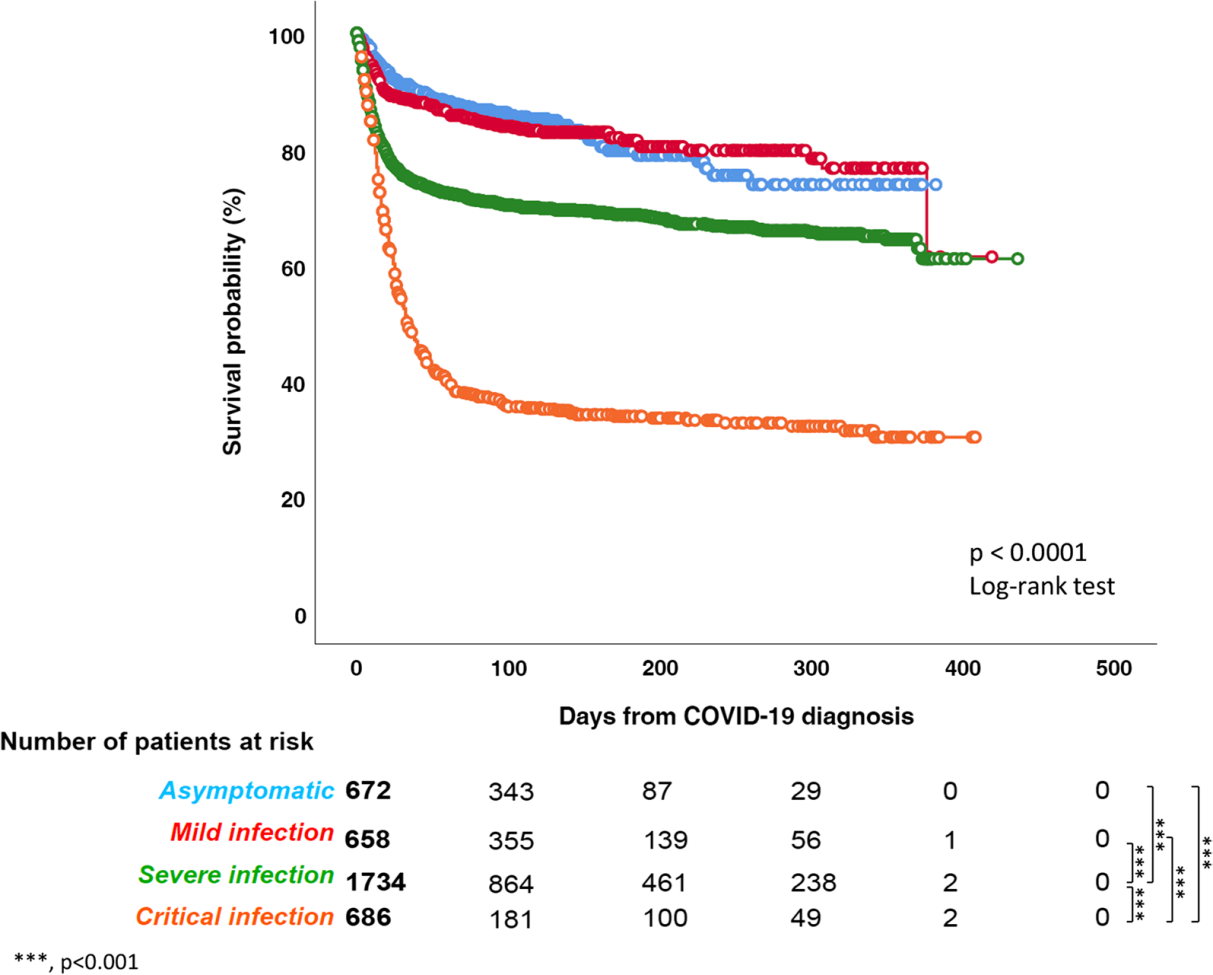
Overall survival by COVID-19 severity

Patients with critical COVID-19 (63.6% [95% CI 59.9–67.1, 438/689]) died in a higher proportion than those with severe (30.3% [95% CI 28.2–32.5, 526/1736]), *p* < 0.0001 or mild infection (16.7% [95% CI 14.1–19.8, 110/658]), *p* < 0.0001. The mortality rate observed in patients with severe infection 30.3% ([95% CI 28.2–32.5] 526/1736), was significantly higher than reported in patients with mild COVID-19 (16.7% [95% CI 14.1–19.8] 110/658), *p* < 0.0001). The mortality in mildly symptomatic patients was not vastly different from that observed in initially asymptomatic patients: 15.4% ([95% CI 12.9–18.3] 104/675) *p* = 0.516 (Fig. 4, Additional file 3: Table 3). Clinical presentation with pulmonary symptoms was associated with a significantly higher mortality rate versus presentation with extrapulmonary symptomatology alone (mortality rate 876/2285, 38.3% [95% CI 36.4–40.4] vs. 163/742, 22.0% [95% CI 19.1–25.1], *p* < 0.0001).

A higher mortality rate was reported for patients admitted to ICU (438/689, 63.5% [95% CI 59.9–67.1]), compared to non-ICU patients (747/3112, 24% [95% CI 22.5–25.5]) *p* < 0.0001. Furthermore, among the ICU patients, a significantly higher mortality rate was observed in patients with invasive MV versus those without (322/449, 71.7% [95% CI 67.4–75.7] vs. 116/240, 48.3% [95% CI 42.1–54.6] *p* < 0.0001).

**Fig. 5:**
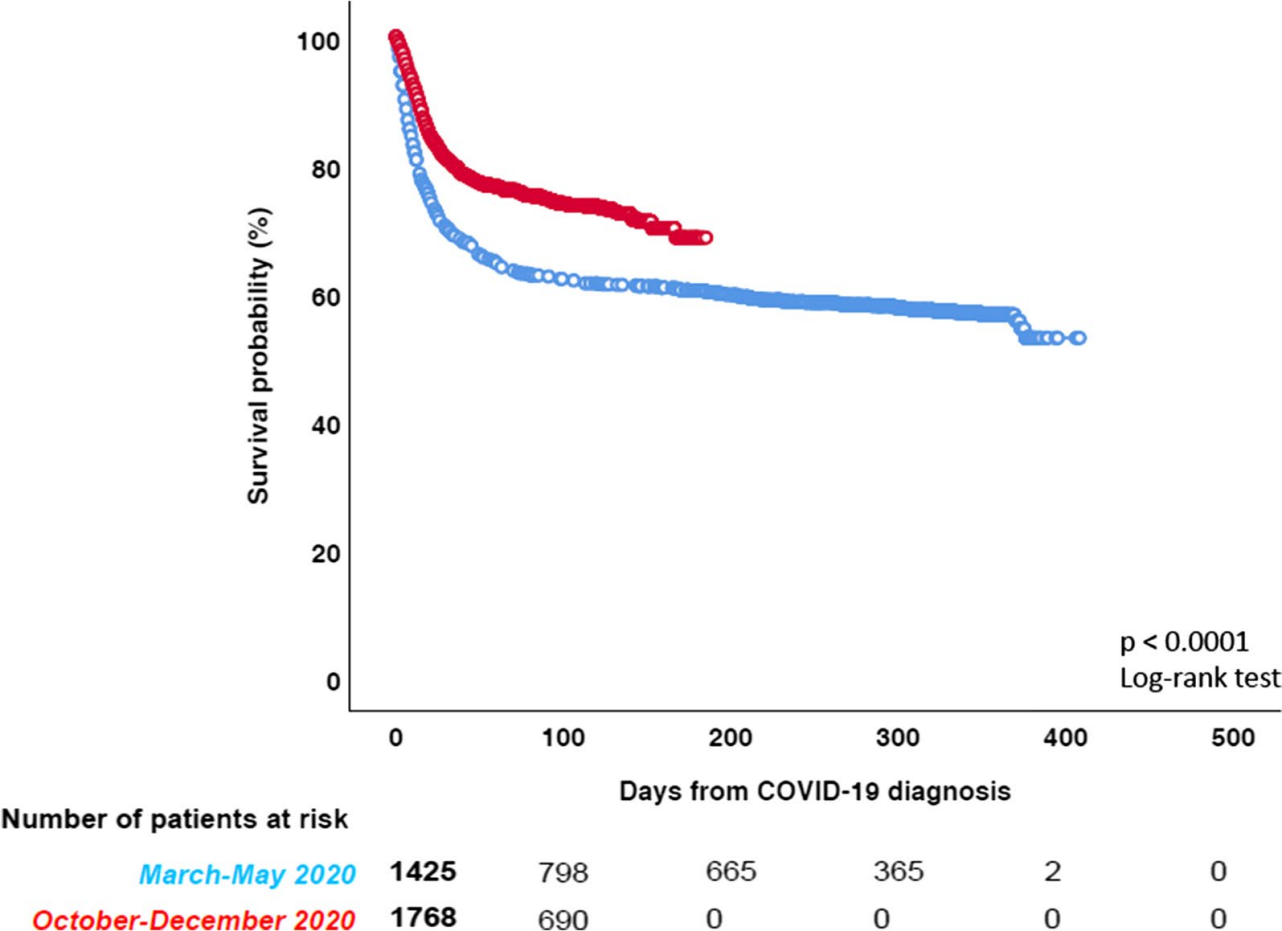
Overall survival by time distribution (first vs. the second wave)

**Fig. 6:**
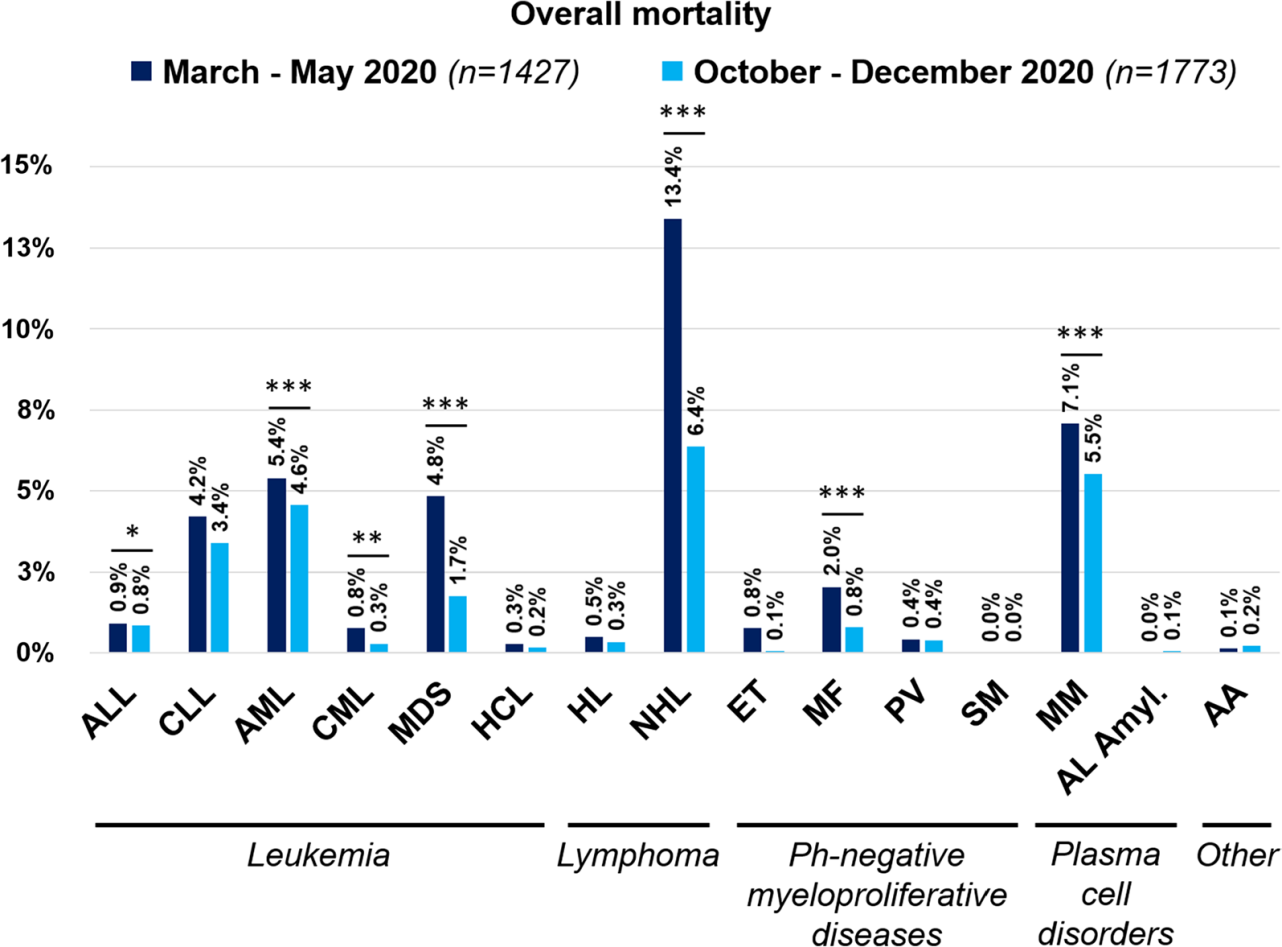
Overall survival in the different HMS by time distribution (first vs. the second wave)

Considering the two waves of COVID-19 (1^st^ wave March–May 2020, 2nd wave October-December 2020), there was a significant decrease in the mortality rate in the second wave (581/1427, 40.7% [95% CI 38.2–43.3] vs. 439/1773, 24.8% [95% CI 22.8–26.8] *p* < 0.0001) (Fig. 5). The reduction of mortality was consistent across different HM diagnoses (Fig. 6).

In the univariable Cox regression analysis, multiple factors negatively influenced mortality (Table 5). Conversely, having a neutrophil count greater than 0.5 × 10^9^/mm^3^ or a lymphocyte count greater than 0.2 × 10^9^/mm^3^ were found to be protective.

**Table 5:** Overall mortality predictors in COVID-19 HM patients

	Univariable			Multivariable		
	*p* value	HR	95% CI	*p* value	HR	95% CI
*Sex*						
Female	–	–	–	–	–	–
Male	**0.059**	1.119	0.095–1.258	0.376	1.065	0.927–1.223
Age	** < 0.0001**	1.036	1.031–1.041	** < 0.0001**	1.032	1.026–1.039
*Malignancy status*						
Controlled disease	–	–	–	–	–	–
Active disease	** < 0.0001**	2.107	1.863–2.383	** < 0.0001**	1.860	1.615–2.141
Unknown	** < 0.0001**	2.293	1.607–3.274	** < 0.0001**	2.353	1.538–3.601
*Hematological malignancy*						
Hodgkin lymphoma	–	–	–	–	–	–
Chronic lymphoid leukemia	** < 0.0001**	2.789	1.635–4.757	0.763	1.093	0.614–1.947
Acute myeloid leukemia	** < 0.0001**	4.364	2.581–7.376	**0.011**	2.046	1.176–3.557
Chronic myeloid leukemia	0.915	0.963	0.481–1.928	0.086	0.513	0.239–1.099
Acute lymphoblastic leukemia	**0.002**	2.530	1.405–4.553	0.250	1.457	0.767–2.768
Non-Hodgkin lymphoma	** < 0.0001**	3.041	1.814–5.100	0.569	1.171	0.68–2.015
Aplastic anemia	**0.040**	2.695	1.045–6.948	0.179	2.022	0.724–5.645
Essential thrombocythemia	0.234	1.585	0.742–3.387	0.332	0.667	0.295–1.511
Multiple myeloma	** < 0.0001**	3.355	1.989–5.658	0.630	1.145	0.661–1.984
Myelodysplastic syndrome	** < 0.0001**	4.627	2.704–7.919	0.072	1.706	0.953–3.056
Myelofibrosis	** < 0.0001**	3.786	2.110–6.791	0.185	1.540	0.813–2.915
Polycythemia vera	0.059	2.016	0.973–4.176	0.985	0.992	0.456–2.158
Amyloidosis	0.893	1.150	0.152–8.705	0.932	-	-
Hairy cell leukemia	**0.019**	2.936	1.197–7.202	0.301	1.806	0.589–5.533
Systemic mastocytosis	**0.715**	1.457	0.192–11.031	0.968	0.959	0.126–7.323
*COVID-19 infection*						
Asymptomatic	–	–	–	–	–	–
Mild infection	0.545	1.087	0.830–1.422	0.653	1.074	0.786–1.467
Severe infection	** < 0.0001**	2.127	1.722–2.628	** < 0.0001**	1.682	1.312–2.157
Critical infection	** < 0.0001**	5.333	4.300–6.613	** < 0.0001**	4.230	3.294–5.432
Unknown	0.623	1.229	0.540–2.800	0.928	–	–
Chronic cardiopathy	** < 0.0001**	2.011	1.792–2.257	** < 0.0001**	1.406	1.218–1.624
Liver disease	**0.008**	1.394	1.091–1.781	**0.020**	1.388	1.052–1.831
Chronic pulmonary disease	** < 0.0001**	1.516	1.320–1.740	0.926	1.008	0.85–1.195
Diabetes mellitus	** < 0.0001**	1.352	1.172–1.560	0.439	1.070	0.901–1.272
Obesity	0.796	0.974	0.796–1.191	–	–	–
Renal impairment	** < 0.0001**	1.883	1.589–2.232	** < 0.0001**	1.404	1.143–1.724
Smoking history	**0.013**	1.224	1.043–1.436	**0.031**	1.223	1.019–1.469
*Neutrophils, cells/mm*^*3*^						
≤ 0.5 × 10^9^/mm^3^	–	–	–	–	–	–
0.501-0.999 × 10^9^/mm^3^	** < 0.0001**	0.594	0.450–0.785	0.272	0.845	0.626–1.141
≥ 1 × 10^9^/mm^3^	** < 0.0001**	0.514	0.431–0.614	0.184	0.862	0.693–1.073
*Lymphocytes, cells/mm*^*3*^						
≤ 0.2 × 10^9^/mm^3^	–	–	–	–	–	–
0.201-0.499 × 10^9^/mm^3^	**0.004**	0.746	0.611–0.912	**0.021**	0.779	0.629–0.963
≥ 0.5 × 10^9^/mm^3^	** < 0.0001**	0.499	0.422–0.590	** < 0.0001**	0.601	0.499–0.722
*Last chemotherapy*						
> 3 months before COVID-19	–	–	–	–	–	–
In the last 3 months	** < 0.0001**	1.531	1.226–1.912	0.081	1.236	0.974–1.568
In the last month	** < 0.0001**	1.688	1.408–2.024	0.657	1.047	0.854–1.284
Unknown	0.103	1.578	0.911–2.734	0.998	0.999	0.537–1.86

*HR* Hazard ratio, *CI* confidence intervals

In the multivariable analysis the following parameters were significantly associated with higher mortality: age increase, active disease, chronic cardiopathy, liver disease, renal impairment, smoking history, and ICU stay. Among HM, AML is the malignancy associated with a significantly high mortality (Table 5).

## Discussion

The incidence of COVID-19 infection in HM ranges between 1 and 3.9% [23]. Mostly, patients get infected in the community, although in 1.1% to 15% of infections nosocomial transmissions are reported [24]. A clear correlation between the type of HM and the incidence of COVID-19 infection has not been described in the literature, but current data indicate that lymphoproliferative disorders, in particular NHL, chronic lymphocytic leukemia, and MM are particularly associated with higher risk from COVID-19.

Here we presented a large survey on COVID-19 among HM patients, with almost 4000 patients reported from 132 hematology institutions mainly located in Europe. In addition, this survey has collected COVID-19 cases from March to December 2020, allowing us to analyze not only which patients were at risk, but also how the infectious process has evolved over time. Our data confirm that a larger number of COVID-19 cases was diagnosed among patients with lymphoproliferative disorders, in particular NHL and MM, as previously documented [9, 10]. However, we also observed a high number of COVID-19 among patients with AML (12.5%), which is considered a rare malignancy. As for comorbidities, our patient population reflects the overall population, with cardiovascular diseases being the most frequent comorbidity reported [16]. Most of the patients recorded in our survey had a severe/critical clinical presentation of COVID-19 (about 60%), over two-thirds were hospitalized and about 18% required ICU admission. These data are not surprising and emphasize the frailty of HM patients, and are slightly higher compared with those reported in the literature, ranging between 15.5 to 52.4% and 6.9 to 14% for severe and critical clinical presentation, respectively [3–17].

The overall and the attributable mortality rates observed in our study (31.2% and 22.2%, respectively) are within the range of those reported in the literature among HM (published reports are summarized in Additional file 4: Table 4), confirming that COVID-19 mortality is significantly higher in HM patients than in the overall population, where current data show a mortality rate ranging between 0.1 and 9.4% across the different countries around the world (www.coronavirus.jhu.edu/data/mortality). Moreover, as expected, the overall mortality rate has been age-dependent, with higher mortality rates observed among patients aged over 70 years. In line with other studies [7, 14, 16], our data have shown that AML and MDS patients, especially those with high-risk MDS, have the worst clinical outcome and the highest mortality rate (up to 45%). In fact, AML was the only that was independently associated with mortality in our multivariable model. A recently published study focusing only on AML patients reported an overall mortality very similar to that described in our study [25]. There are several possible explanations of this phenomenon. First, patients with AML/MDS are often aged over than 65 years old. Second, they present a profound immunodeficiency as a consequence of both disease and treatments received. Third, they are patients in which a treatment delay is often not possible due to the urgent need of starting an active therapy. This last aspect is quite relevant, especially if we consider that a lower mortality in patients who delayed AML treatment was described compared to those with and without treatment modification [25]. In high-risk MDS patients, treatment with demethylating agents was associated with a particularly high mortality rate. Our study highlights the role of these agents as being potentially associated with high mortality in AML/MDS patients with COVID-19. Our data also showed that patients undergoing HSCT (either autologous or allogeneic) presented a significantly lower mortality rate following COVID-19, compared to non-transplant patients. We report an overall mortality rate of 24.8% and 27% in allo-HSCT and auto-HSCT, respectively, almost identical to that very recently described in the study of the European Society for Blood and Marrow Transplantation [12]. This observation is coherent with previous published data, suggesting a significantly lower mortality rate among transplanted patients compared with non-transplanted HM patients [15]. Patients who receive HSCT, especially an allogeneic one, are by definition younger and healthier than the overall onco-hematological patients. In fact, we observed that most of conditions associated with higher overall mortality (i.e. older age, comorbidities, uncontrolled disease) were overrepresented in the non-transplant cohort. These aspects may explain in part the lower mortality we observed in transplanted patients. Interestingly, patients undergoing CAR-T infusion have shown a worse clinical outcome in our survey, with 10 deaths among 21 COVID-19 patients registered in the database. Other significant predictors of mortality in the multivariable analysis included active disease, chronic cardiopathy, liver disease, renal impairment, smoking history, and ICU stay.

Moreover, we found a significantly lower mortality in COVID-19 HM patients in the second wave as compared to the first wave of COVID 19. Improved clinical outcome has been documented for many different diseases, including those with the highest mortality rates. This improvement in the second wave of COVID-19 is of interest, and could be the result of several factors, including a better knowledge of the clinical course of the disease, more effective protective procedure for HM patients, a detection of a larger number of asymptomatic/mild cases by screening swabs and/or an improvement of specific treatments against COVID-19, for example remdesivir, monoclonal antibodies, convalescent plasma. Coherently with our hypothesis, in the second wave, we found a significantly higher rate of asymptomatic and mild infections and a significantly lower rate of severe infections. However, even though we did not observe significant differences in HM distribution, in the second wave we found more patients with controlled disease compared to the first one.

We strongly believe that our findings will impact the management of HM patients also in the near future. Even if we are witnessing a huge worldwide vaccination program, preliminary data published so far suggest that anti-SARS-CoV-2 vaccines shows significantly less robust efficacy in eliciting an immune response in HM patients than observed in the general population [26, 27]. Moreover, we are assisting to the wide diffusion of variants of concerns under the vaccine selective pressure. Indeed, several cases of breakthrough infections have been reported in the general population, with a significant mortality rate [28, 29]. We expect that, in the immediate future, we will assist to several cases of SARS-CoV-2 infections in fully vaccinated HM patients. From this point of view, the better understanding of epidemiologic features and risk factors for COVID-19 in HM patients, might surely help hematologists in the management of their patients and even in modifying the chemotherapeutic programs where possible. HM patients still deserve special attention and protective measures should continue.

Our large registry study comes with some limitations. First, at the time the study was designed, the role of thromboembolic phenomena of COVID-19 infection was still unknown and therefore not included in the survey. Second, we have deliberately excluded the data relating to the various COVID-19 therapeutic approaches because they are extremely heterogeneous and treatment recommendations change rapidly. Third, due to our registry design we have not been able to calculate the incidence of COVID-19 in the various subclasses of HM. Last, due to the intrinsic limitations of the study, it is not possible to provide cumulative incidences regarding relevant aspects, such as mortality, as there is no certainty about whether all participating sites documented all eligible cases.

These data need to be carefully interpreted considering the incidence of individual HM in the general population and the patient performance status, which affects their social dimension and lifestyle in the community.

## Conclusion

This study sheds light on the epidemiology, risk factors and outcomes of COVID-19 among patients with HM. While the introduction of COVID-19 vaccinations will lead to a marked reduction of infections in HM patients, the possibility of a lower efficacy of vaccinations needs to be taken into account [30], possibly resembling previous experiences with influenza vaccination. Future studies are needed to evaluate whether the use of vaccination will be able to prevent the development and above all mortality in the identified risk categories of HM.

## Supplementary Information

**Additional file 1: Supplementary Table 1.** Partnership from National and International Scientific Society.

**Additional file 2: Supplementary Table 2.** List of participating institutions.

**Additional file 3: Supplementary Table 3.** Demographic and clinical characteristics of enrolled patients depending on the COVID-19 severity.

**Additional file 4: Supplementary Table 4.** Multicentre studies on COVID-19 in patients with haematologic malignancies reported during 2020.

## Data Availability

Individual participant data that underlie the results reported in this Article, after de-identification (text, tables, figures, and appendices), will be available together with the study protocol. This will be from 9 to 24 months following Article publication. Data will be available only for investigators whose proposed use of the data has been approved by an independent review committee identified for this purpose.

## Abbreviations

allo-HSCT: Allogeneic HSCT [hematopoietic stem cell transplantation]
AML: Acute myeloid leukemia
ASH: American Society of Hematology
auto-HSCT: Autologous HSCT [hematopoietic stem cell transplantation]
BAL: Bronchoalveolar lavage
CAR-T: Chimeric antigen receptor T-cell therapies
CI: Confidence intervals
COVID-19: Coronavirus disease 19
eCRF: Electronic case report form
EHA: European Hematology Association
EPICOVIDEHA: Epidemiology of COVID-19 Infection in Patients with Hematological Malignancies: A European Hematology Association Survey
HM: Hematological malignancy
HR: Hazard ratio
HSCT: Hematopoietic stem cell transplantation
ICU: Intensive care unit
IQR: Interquartile range
MDS: Myelodysplastic syndromes
MM: Multiple myeloma
MV: Mechanical ventilation
NHL: Non-Hodgkin lymphoma
SARS-CoV-2: Severe acute respiratory syndrome coronavirus 2
TKI: Tyrosine kinase inhibitors
WHO: World Health Organization
