# Supplementary material for: COVID-19 infection in adult patients with hematological malignancies: a European Hematology Association Survey (EPICOVIDEHA)
Source: J Hematol Oncol. 2021 Oct 14;14:168. doi: 10.1186/s13045-021-01177-0 (PMC8515781; doi:10.1186/s13045-021-01177-0)
Supplement: Supplementary file 1 — Additional file 1: Supplementary Table 1. Partnership from National and International Scientific Society. [file 13045_2021_1177_MOESM1_ESM.docx]

**Supplementary Table 1**: Partnership from National and International Scientific Society

- EHA- IDWG Infectious Diseases
- EHA-SWG Aging and Hematology
- Supportive Treatment Group of the Croatian Cooperative Group for Hematological Diseases
- SEIFEM Group (Sorveglianza Epidemiologica InFezioni nelle Emopatie)
- Arbeitsgemeinschaft Infektionen in der Hämatologie und Onkologie [AGIHO] of the Deutschen Gesellschaft für Hämatologie und Onkologie [DGHO]
- Danish National Registry of COVID-19
- SIE (Società Italiana di Ematologia)
- CELL (Czech Leukemia Study Group – for Life)
- SEHH (Societad Espanola de Hematologia y Hemoterapia)
- Israel Hematology Association
