# Supplementary material for: COVID-19 infection in adult patients with hematological malignancies: a European Hematology Association Survey (EPICOVIDEHA)
Source: J Hematol Oncol. 2021 Oct 14;14:168. doi: 10.1186/s13045-021-01177-0 (PMC8515781; doi:10.1186/s13045-021-01177-0)
Supplement: Supplementary file 3 — Additional file 3: Supplementary Table 3. Demographic and clinical characteristics of enrolled patients depending on the COVID-19 severity. [file 13045_2021_1177_MOESM3_ESM.docx]

**Supplementary Table 3**: Demographic and clinical characteristics of enrolled patients depending on the COVID-19 severity.

|  | **Asymptomatic** | | **Mild infection** | | **Severe infection** | | **Critical infection** | | **Unknown** | |
| --- | --- | --- | --- | --- | --- | --- | --- | --- | --- | --- |
|  | n | % | n | % | n | % | n | % | n | % |
| **Sex** |  |  |  |  |  |  |  |  |  |  |
| Female | 304 | 45.0% | 297 | 45.1% | 689 | 39.7% | 275 | 39.9% | 14 | 32.6% |
| Male | 371 | 55.0% | 361 | 54.9% | 1047 | 60.3% | 414 | 60.1% | 29 | 67.4% |
| **Age**, median (IQR) [range] | 61 (48 - 70), [18 - 93] | | 63 (51 - 74), [18 - 95] | | 69 (57 - 77), [18 - 94] | | 64 (55 - 72), [18 - 92] | | 59 (48 - 72), [22 - 87] | |
| **Ethnic origin** |  |  |  |  |  |  |  |  |  |  |
| American Indian/Alaska Native | 0 | 0.0% | 0 | 0.0% | 2 | 0.1% | 0 | 0.0% | 0 | 0.0% |
| Asian | 12 | 1.8% | 6 | 0.9% | 51 | 2.9% | 14 | 2.0% | 0 | 0.0% |
| Black/African American | 5 | 0.7% | 4 | 0.6% | 19 | 1.1% | 4 | 0.6% | 1 | 2.3% |
| Pacific Islander/Native Hawaiian | 0 | 0.0% | 0 | 0.0% | 0 | 0.0% | 0 | 0.0% | 0 | 0.0% |
| White | 627 | 92.9% | 586 | 89.1% | 1476 | 85.0% | 563 | 81.7% | 32 | 74.4% |
| Unknown | 31 | 4.6% | 62 | 9.4% | 188 | 10.8% | 108 | 15.6% | 10 | 23.3% |
| **Comorbidities** ° |  |  |  |  |  |  |  |  |  |  |
| Chronic cardiopathy | 154 | 22.8% | 174 | 26.4% | 583 | 33.6% | 224 | 32.5% | 11 | 25.6% |
| Chronic pulmonary disease | 51 | 7.6% | 79 | 12.0% | 343 | 19.8% | 135 | 19.6% | 6 | 14.0% |
| Diabetes mellitus | 94 | 13.9% | 94 | 14.3% | 314 | 18.1% | 115 | 16.7% | 3 | 7.0% |
| Liver disease | 23 | 3.4% | 23 | 3.5% | 84 | 4.8% | 36 | 5.2% | 1 | 2.3% |
| Obesity | 65 | 9.6% | 49 | 7.4% | 139 | 8.0% | 88 | 12.8% | 4 | 9.3% |
| Renal impairment | 43 | 6.4% | 53 | 8.1% | 164 | 9.4% | 61 | 8.9% | 4 | 9.3% |
| Smoking history | 87 | 12.9% | 74 | 11.2% | 215 | 12.4% | 98 | 14.2% | 3 | 7.0% |
| No risk factor identified | 321 | 47.6% | 290 | 44.1% | 601 | 34.6% | 233 | 33.8% | 18 | 41.9% |
| **Underlying malignancy** |  |  |  |  |  |  |  |  |  |  |
| Leukemia | 262 | 38.8% | 258 | 39.2% | 746 | 43.0% | 318 | 46.2% | 19 | 44.2% |
| Acute lymphoid leukemia | 47 | 7.0% | 31 | 4.7% | 50 | 2.9% | 39 | 5.7% | 2 | 4.7% |
| Chronic lymphoid leukemia | 45 | 6.7% | 66 | 10.0% | 263 | 15.1% | 97 | 14.1% | 3 | 7.0% |
| Acute myeloid leukemia | 105 | 15.6% | 80 | 12.2% | 188 | 10.8% | 120 | 17.4% | 4 | 9.3% |
| Chronic myeloid leukemia | 29 | 4.3% | 32 | 4.9% | 77 | 4.4% | 17 | 2.5% | 6 | 14.0% |
| Myelodisplastic syndrome | 35 | 5.2% | 48 | 7.3% | 157 | 9.0% | 36 | 5.2% | 3 | 7.0% |
| *Low - intermediate risk* | 14 | 2.1% | 26 | 4.0% | 85 | 4.9% | 12 | 1.7% | 1 | 2.3% |
| *High risk* | 6 | 0.9% | 5 | 0.8% | 29 | 1.7% | 8 | 1.2% | 0 | 0.0% |
| *Not stated* | 15 | 2.2% | 17 | 2.6% | 43 | 2.5% | 16 | 2.3% | 2 | 4.7% |
| Hairy cell leukemia | 1 | 0.1% | 1 | 0.2% | 11 | 0.6% | 9 | 1.3% | 1 | 2.3% |
| Lymphoma | 238 | 35.3% | 228 | 34.7% | 528 | 30.4% | 211 | 30.6% | 14 | 32.6% |
| Hodgkin lymphoma | 39 | 5.8% | 30 | 4.6% | 50 | 2.9% | 11 | 1.6% | 5 | 11.6% |
| Non-Hodgkin lymphoma | 199 | 29.5% | 198 | 30.1% | 478 | 27.5% | 200 | 29.0% | 9 | 20.9% |
| *Indolent* | 64 | 9.5% | 84 | 12.8% | 244 | 14.1% | 103 | 14.9% | 2 | 4.7% |
| *Aggressive* | 120 | 17.8% | 103 | 15.7% | 209 | 12.0% | 80 | 11.6% | 4 | 9.3% |
| *Not stated* | 15 | 2.2% | 11 | 1.7% | 25 | 1.4% | 17 | 2.5% | 3 | 7.0% |
| PH negative myeloproliferative diseases | 39 | 5.8% | 59 | 9.0% | 125 | 7.2% | 38 | 5.5% | 6 | 14.0% |
| Essential thrombocythemia | 12 | 1.8% | 16 | 2.4% | 32 | 1.8% | 5 | 0.7% | 4 | 9.3% |
| Myelofibrosis | 15 | 2.2% | 22 | 3.3% | 63 | 3.6% | 21 | 3.0% | 1 | 2.3% |
| Polycythemia vera | 10 | 1.5% | 21 | 3.2% | 27 | 1.6% | 11 | 1.6% | 1 | 2.3% |
| Systemic mastocytosis | 2 | 0.3% | 0 | 0.0% | 3 | 0.2% | 1 | 0.1% | 0 | 0.0% |
| Plasma cell disorders | 133 | 19.7% | 105 | 16.0% | 331 | 19.1% | 120 | 17.4% | 3 | 7.0% |
| Multiple myeloma | 131 | 19.4% | 101 | 15.3% | 330 | 19.0% | 119 | 17.3% | 3 | 7.0% |
| Amyloid light-chain amyloidosis | 2 | 0.3% | 4 | 0.6% | 1 | 0.1% | 1 | 0.1% | 0 | 0.0% |
| Other hematological malignancies | 3 | 0.4% | 8 | 1.2% | 6 | 0.3% | 2 | 0.3% | 1 | 2.3% |
| Aplastic anemia | 3 | 0.4% | 8 | 1.2% | 6 | 0.3% | 2 | 0.3% | 1 | 2.3% |
| **Last/ongoing treatment**  **strategy before COVID-19** |  |  |  |  |  |  |  |  |  |  |
| Anagrelide/Hydroxyurea | 21 | 3.1% | 30 | 4.6% | 69 | 4.0% | 22 | 3.2% | 3 | 7.0% |
| Conventional chemotherapy | 139 | 20.6% | 110 | 16.7% | 198 | 11.4% | 121 | 17.6% | 4 | 9.3% |
| Demethylating agents | 19 | 2.8% | 21 | 3.2% | 72 | 4.1% | 28 | 4.1% | 1 | 2.3% |
| Immunotherapy only | 21 | 3.1% | 25 | 3.8% | 54 | 3.1% | 23 | 3.3% | 2 | 4.7% |
| Immunochemotherapy | 149 | 22.1% | 145 | 22.0% | 383 | 22.1% | 171 | 24.8% | 9 | 20.9% |
| IMIDs | 37 | 5.5% | 32 | 4.9% | 114 | 6.6% | 35 | 5.1% | 0 | 0.0% |
| Targeted therapy | 105 | 15.6% | 103 | 15.7% | 291 | 16.8% | 100 | 14.5% | 8 | 18.6% |
| Palliative | 26 | 3.9% | 21 | 3.2% | 68 | 3.9% | 36 | 5.2% | 0 | 0.0% |
| Maintenance | 4 | 0.6% | 5 | 0.8% | 11 | 0.6% | 4 | 0.6% | 1 | 2.3% |
| Other | 6 | 0.9% | 5 | 0.8% | 9 | 0.5% | 8 | 1.2% | 0 | 0.0% |
| HSCT - Allogeneic | 44 | 6.5% | 33 | 5.0% | 66 | 3.8% | 29 | 4.2% | 1 | 2.3% |
| HSCT - Autologous | 23 | 3.4% | 16 | 2.4% | 21 | 1.2% | 14 | 2.0% | 0 | 0.0% |
| CAR-T | 2 | 0.3% | 3 | 0.5% | 8 | 0.5% | 7 | 1.0% | 1 | 2.3% |
| Radiotherapy | 1 | 0.1% | 0 | 0.0% | 6 | 0.3% | 2 | 0.3% | 1 | 2.3% |
| Unknown | 12 | 1.8% | 9 | 1.4% | 15 | 0.9% | 5 | 0.7% | 0 | 0.0% |
| No treatment | 61 | 9.0% | 81 | 12.3% | 303 | 17.5% | 83 | 12.0% | 10 | 23.3% |
| Supportive measures | 5 | 0.7% | 19 | 2.9% | 48 | 2.8% | 1 | 0.1% | 2 | 4.7% |
| **Overall mortality** | 104 | 15.4% | 110 | 16.7% | 526 | 30.3% | 438 | 63.6% | 7 | 16.3% |
| Reason for death° |  |  |  |  |  |  |  |  |  |  |
| Not related to COVID-19 | 36 | 5.3% | 32 | 4.9% | 73 | 4.2% | 46 | 6.7% | 0 | 0.0% |
| Contributable by COVID-19 | 26 | 3.9% | 17 | 2.6% | 54 | 3.1% | 55 | 8.0% | 3 | 7.0% |
| Attributable to COVID-19 | 42 | 6.2% | 61 | 9.3% | 399 | 23.0% | 337 | 48.9% | 4 | 9.3% |
| Attributable to hematological malignancy | 51 | 7.6% | 40 | 6.1% | 136 | 7.8% | 100 | 14.5% | 1 | 2.3% |
| Death due to other reasons | 11 | 1.6% | 12 | 1.8% | 56 | 3.2% | 40 | 5.8% | 1 | 2.3% |
| Death due to unknown reasons | 7 | 1.0% | 8 | 1.2% | 24 | 1.4% | 33 | 4.8% | 0 | 0.0% |

° Data can be super additive; ^ Data not available in all patients.

**HSCT:** haematopoietic stem cell transplantation; **CAR-T:** chimeric antigen receptor T-cell therapies.
