# Supplementary material for: COVID-19 infection in adult patients with hematological malignancies: a European Hematology Association Survey (EPICOVIDEHA)
Source: J Hematol Oncol. 2021 Oct 14;14:168. doi: 10.1186/s13045-021-01177-0 (PMC8515781; doi:10.1186/s13045-021-01177-0)
Supplement: Supplementary file 4 — Additional file 4: Supplementary Table 4. Multicentre studies on COVID-19 in patients with haematologic malignancies reported during 2020. [file 13045_2021_1177_MOESM4_ESM.docx]

| Supplementary table 5. Multicentre studies on COVID-19 in patients with haematologic malignancies reported during 2020 | | | | | | | | | | |
| --- | --- | --- | --- | --- | --- | --- | --- | --- | --- | --- |
|  | **Kind of Study/Country** | **AML**  **cases/deaths** | **MDS**  **cases/deaths** | **CMD**  **cases/deaths** | **NHL and HL**  **cases/deaths** | **MM**  **cases/deaths** | **ALL**  **cases/deaths** | **CLL**  **cases/deaths** | **Others**  **cases/deaths** | **Total**  **cases/deaths** |
| Passamonti et al.  Lancet Hematol 2020 | Italy | 51/22  (43%) | 41/20  (49%) | 83/27  (33%) | 170/65(  38%) | 106/39  (37%) | 16/3  (19%) | 69/22  (32%) | - | 536/198  (37%) |
| Yigenoglu et al.  Med Virol 2020 | Turkey | 40/8  (20%) | 146/22  (15%) | 146/13  (9%) | 250/28  (11%) | 77/15  (19.5%) | 18/3  (17%) | 54/9  (17%) | 9/4  (44%) | 740/102  (13.8%) |
| Garcia Suarez et al.  Hematol Oncol 2020 | Spain | 61/27  (44%) | 78/33  (42%) | 79/14  (18%) | 220/68  (31%) | 137/47  (34%) | 13/2  (15%) | 109/39  (36%) | - | 697/230  (33%) |
| Glentoj et al.  Eur J Haematol 2020 | Denmark | 8/5  (63%) | - | - | - | 11/2  (18%) | - | 31/6  (19%) | 16/3  (19%) | 66/16  (24%) |
| Lee et al.  Lancet Oncol 2020 | UK | 79/33  (42%) | - | - | 79/25  (32%) | 37/16  (43%) | - | - | 29/7  (24%) | 224/81  (36%) |
| Pinana et al.  Exp Hemat Oncol 2020 | Spain | 67 | 22 | 29 | 91 | 81 | 25 | 4 | 19 | 338/105  (31%) |
| Kuderer et al.  Lancet 2020 | Global | 13 | - | - | 81 | 55 | 6 | - | 49 | 204/24  (11.8%) |
| Wood et al.  Blood Adv 2020 | Global | 82/26  (32%) | - | 24/6  (25%) | 79/20  (25%) | 40/11  (28%) | - | 29/8  (28%) | - | 254/71  (28%) |
| Borah et al.  Blood Cell and Mol Dis 2021 | India | 24/8  (33%) | 11/1  (7%) | 4/0  (0%) | 37/8  (21.6%) | 23/5  (21.6%) | 31/4  (14%) | - | - | 130/26  (20%) |
| Cattaneo et al. Cancer 2020 | Italy | 8/4  (50%) | 6/4  (67%) | 10/5  (50%) | 42/17  (41%) | 18/5  (28%) | - | 11/3  (27%) | 7/2  (29%) | 102/40  (39%) |
| ASH research collaborative 2021^ | Global registry data summaries | 337/57  (16.9%) | - | 103/12  (11.6%) | 259/48  (18.5%) | 207/30  (14.5%) | - | 119/20  (16.8%) | - | 1013/165  (16.3%) |
| Totals/deaths |  |  |  |  |  |  |  |  |  | **4304/1058**  **(24.6%)** |

AML, acute myeloid leukaemia; MDS, myelodysplastic syndrome; CMD, chronic myeloproliferative diseases; NHL, non-Hodgkin lymphoma; HL, Hodgkin lymphoma; MM, multiple myeloma; ALL, acute lymphatic leukaemia; CLL, chronic lymphatic leukaemia. ^ Data updated at June 2021.
